# Potential Impact of B Cells on T Cell Function in Multiple Sclerosis

**DOI:** 10.1155/2011/423971

**Published:** 2011-03-24

**Authors:** Sara Ireland, Nancy Monson

**Affiliations:** Departments of Neurology, Neurotherapeutics and Immunology, University of Texas Southwestern Medical Center, Dallas, TX 75390, USA

## Abstract

Multiple sclerosis is a chronic debilitating autoimmune disease of the central nervous system. The contribution of B cells in the pathoetiology of MS has recently been highlighted by the emergence of rituximab, an anti-CD20 monoclonal antibody that specifically depletes B cells, as a potent immunomodulatory therapy for the treatment of MS. However, a clearer understanding of the impact B cells have on the neuro-inflammatory component of MS pathogenesis is needed in order to develop novel therapeutics whose affects on B cells would be beneficial and not harmful. Since T cells are known mediators of the pathology of MS, the goal of this review is to summarize what is known about the interactions between B cells and T cells, and how current and emerging immunotherapies may impact B-T cell interactions in MS.

## 1. Introduction

It has long been established that T cells are mediators of the pathology of multiple sclerosis (MS) in both murine models and patient studies [1–6]. Although, the impact of B cells and their antibody products in mediating the pathology of MS has long been considered [7–10], their contributions have been more recently highlighted by the demonstration that Rituximab, an anti-CD20 monoclonal antibody that specifically depletes B cells, was a potent immunomodulatory therapy for the treatment of MS [11, 12]. More importantly, however, the efficacy of Rituximab in the treatment of MS patients is independent of secreted antibody since Rituximab does not affect plasma cell frequencies or serum and cerebrospinal fluid (CSF) antibody levels [13]. Thus, scientists in the field have refocused their attention on the possible roles of B cells in MS that are independent of their antibody secreting function. This paper summarizes the possible “antibody secretion-independent” roles of B cells on T cell activation and regulation, the relative impact of the B cell subpopulations on T cell activation and regulation, evidence that these mechanisms are altered in MS, and how current and emerging immunotherapies may impact B-T cell interactions in MS.

## 2. What Is known about the Consequences of B-T Cell Interactions?

It has long been assumed that B cells are unlikely to play a significant role as antigen-presenting cells (APCs) in the induction of effector T cells since human B cells are less potent APCs *in vitro* on a per-cell basis compared to dendritic cells [14]. However, in 1982, investigators published for the first time that human B cells could present antigens [15]. In fact, B cells are potent APCs in humans *in vitro* in the context of both alloantigen [16, 17] and exogenous-foreign-antigen [18] responses. Studies in mouse models in which the B cells cannot secrete antibodies have further highlighted the importance of antibody independent B cell responses [19, 20]. These results demonstrated that B cells are required for generating optimal primary and secondary T cell responses and are implicated as APC in a number of disease models in the mouse including rheumatoid arthritis and type 1 diabetes [21–23].

More recently, it has been demonstrated that activated B cells are more effective in activating T cells than their resting or naïve counterparts in the mouse [24–26]. This finding has been confirmed with human B cells as well, since human naïve B cell alloantigen presentation can be increased with CpG-ODN stimulation [27]. Antigen-specific B cell APC function can also be increased with CD40L stimulation [27–31]. 

The most well-studied consequence of B-T cell interactions, however, is the induction of T cell tolerance or expansion of regulatory T cells [32–34]. For example, in mice, antigen specific naïve B cells induce naïve T cells to proliferate and differentiate into regulatory T cells [35]. HEL-specific CD43− (naïve) B cells do not elicit T cell proliferation or IL-2 and interferon-gamma (IFN*γ*) secretion, suggesting that they induce T cell tolerance [36]. In mouse models, naïve B cells did not participate in T cell priming *in vivo*, implicating naïve B cells as a source of regulatory B cells [25, 37, 38]. 

However, few have examined the role of memory B cell antigen presentation in humans, which is a crucial consideration since memory B cells are antigen experienced. Our group demonstrated that memory B cells from healthy donors express sufficient levels of CD80 and HLA-DR to operate as antigen presenting cells to induce antigen specific T cell proliferation and IFN*γ* secretion [39]. Our group has also found that memory B cells from these same healthy donors also produce high concentrations of lymphotoxin- alpha (LT*α*) in comparison to their naïve counterparts. Another group demonstrated that memory B cells from healthy donors induce T cell proliferation in response to glatiramer acetate (GA), a peptide mimic of the neuro-antigen, myelin basic protein (MBP), with some indication that CD80 expression is important for this function [18]. In a separate study [16], it was demonstrated that resting memory B cells from healthy donors elicit robust allogeneic T cell proliferation that was significantly inhibited by anti-CD80 and anti-CD86 antibodies in contrast to naïve B cells, which were unable to stimulate allogeneic T cell proliferation. With the exception of these three studies, memory B cell antigen presentation in healthy donors has not been explicitly examined. 

Memory B cells from treatment naïve RRMS patients, however, have the unique ability to present neuroantigens to autologous T cells and generate a proliferative response and IFN*γ* production [39]. Memory B cells from healthy donors are unable to support T cell proliferation and IFN*γ* production in response to neuroantigens. Taken together it appears that memory B cells might directly contribute to T cell activation by presenting neuro-antigens and secreting cytokines that enhance the Th1/IFN*γ* producing T cell subset. We are currently testing whether memory B cells from treatment-exposed RRMS patients maintain their capacity to incite T cell activation in a neuroantigen specific manner. 

These findings have generated considerable interest in dissecting the mechanism of B-T cell interactions, especially as they relate to the antigen experience of B cells. The two primary antibody secretion independent ways that B cells potentially impact T cell activation or regulation are by (1) providing costimulatory signals through direct B-T cell interactions and (2) cytokine secretion. The following sections detail the primary surface molecules and secreted factors that are thought to contribute to T cell activation or regulation by B cells.

## 3. What Types of Surface Molecules Expressed by B Cells Could Be Influencing T Cell Function?

**Figure 1:**
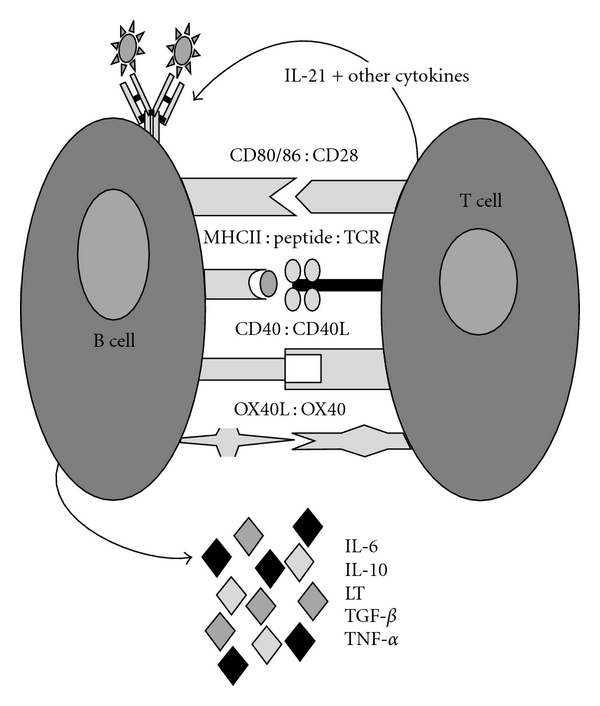
Important co-stimulatory molecules in B-T cell interactions.

Figure 1 depicts aspects of the currently accepted model of important B-T cell interactions [40, 41]. Interactions between B7.1/B7.2 (CD80/CD86) expressed on B cells and their ligands and CD28 and CTLA4 expressed on T cells have long been studied in MS [42, 43], and much is understood regarding the influence of these interactions on T cell responses [44–46]. In fact, circulating CD80+ B cells are increased during active relapse phases of MS, compared to patients in remission or controls [47], suggesting that this population may be involved during the active phase of the disease. Importantly, others have demonstrated that a subset of memory B cells, but not naïve B cells, express CD80 [16, 18] CD86 [16], and CD25, although this expression is not necessarily concurrent [18, 48]. The impact of CD80 expression on B cells and their ability to activate T cells is certainly of interest since the stimulation of memory T cells is CD28 independent [49].

Interactions between MHCII, peptide, and the TCR are certainly central to effective B-T cell costimulation, especially in the context of MS, in which HLA-DR haplotypes have a strong association [50–52]. T cell proliferation in the presence of CD19+ B cells (containing both naïve and memory B cells) activated with CD40L and antigen is reduced by ~60% in the presence of anti-HLA-DR antibodies [28]. 

Interactions between CD40 expressed on B cells and CD40 ligand (CD40L) expressed on T cells are also critical to mount an effective T cell response [53]. This consistent observation led to the development of anti-CD40L biologics to dampen T cell responses but became problematic due to the increase in thromboembolic events associated with these biologics, and the discovery that activated platelets express CD40L [54, 55]. Anti-CD40 agonists, however, are being developed for applications in tumor immunotherapy to mobilize T cells in patients with cancer [56]. Yet it is unclear what *types* of T cells are mobilized by CD40-activated B cells. This is a critical point to consider since purified B cells stimulated with one of these new CD40 agonists in combination with CpG (a TLR9 agonist) secreted high levels of both IL-6 and IL-10 [57, 58]. 

Interactions between OX40L expressed on APCs and OX40 expressed on T cells have profound effects on T cell responses. For example, Wang et al. have reported that co-culturing IFN*γ*-activated microglia from mouse brain tissue with murine CD4+ T cells significantly increased T cell proliferation, which was readily blocked by the addition of anti-OX40L in the cultures [59]. In humans, exposure of human T cells to OX40L prevents the development of IL-10-producing T cells [60] and IL-17 producing T cells [61]. Thus, biologics that block or enhance these interactions may be useful therapeutics for autoimmunity and infection, respectively, and are currently being considered for development in these arenas. However, the impact of OX40-OX40L ligation on B-T cell interactions has not been formally investigated.

## 4. What Types of Secreted Factors Produced by B Cells Could Be Influencing T Cell Function?

The influence of T cell derived cytokines in health and disease has dominated scientific literature for at least 2 decades. However, it has become apparent that activated B cells have a high capacity to produce both inflammatory and regulatory cytokines [92] (Table 1). For example, recent investigations have demonstrated that IL-10-producing B cells have an immunomodulatory role in experimental allergic encephalomyelitis (EAE), which is the mouse model of MS [93, 94]. However, CD19+ B cell pools from MS patients have a diminished capacity to produce IL-10 [95, 96], suggesting that the inflammatory responses in MS patients may be partially attributable to a defect in IL-10 production by B cells. *In vitro* activation of purified naïve and memory B cells from the RRMS patient cohort used in our studies using CD40L (as was done by [96]), demonstrates that naïve B cells produced similar amounts of IL-10 reported by [66, 95, 97], but memory B cells did not produce appreciable amounts of IL-10 [39]. Interestingly, when we cultured naïve B cells with T cells in our B-T cell culture system, we did not observe appreciable T cell proliferation in response to neuro-antigens, suggesting that the amount of IL-10 produced by naïve B cells from RRMS patients may regulate T cell responses. In support of this concept, IL-10 treatment of CD4 T cells from patients with rheumatoid arthritis significantly decreased the numbers of Th17 cells *in vitro* [98]. 

So what *pro*-inflammatory cytokines do B cells generate that have a high likelihood of effecting T cell function in response to neuro-antigens? LT*α* and tumor necrosis factor alpha (TNF-*α*) are classic candidates of the TNF superfamily with similar structure [99, 100]. Their expression by B cells is required for germinal center formation [101] and the migration of antigen-loaded myeloid dendritic cells (mDCs) to follicles [102]. LT*α* is also required for the generation of memory B cells and isotype switch [103]. LT*α* and TNF-*α* are readily produced by activated memory B cells from healthy donors [104], and T cells express receptors for these cytokines [105]. Furthermore, TNF*α* and LT*α* levels are increased in MS lesions and mediate oligodendrocyte toxicity *in vitro *[106]. B cells from RRMS patients showed increased LT*α* and TNF*α* and decreased IL-10 production in response to polyclonal stimuli (CD40L and BCR crosslinking) in the presence of a TLR9 agonist or IFN*γ* [107]. In fact, when CD19+ cells are removed *in vitro* from peripheral blood mononuclear (PBMC) cultures, CD4+ and CD8+ T cell proliferation is decreased and may be due to a lack of B cells secreting LT*α* and TNF*α* [107], which would support T cell proliferation.

Transforming growth factor-beta (TGF-*β*) and IL-6 are readily secreted by activated B cells as well [108], and there is certainly much excitement regarding the ability of TGF*β* and IL-6 in combination to mediate Th17 cell activity since Th17 cells are central to the inflammation associated with EAE [1, 109–114]. However, the classic role of TGF*β* has been to induce tolerance in the immune system, and indeed, anti-TGF*β* treatment of mice led to worsening of EAE disease and more severe clinical pathology in these mice [115]. TGF*β* has been attempted ineffectively as a treatment for progressive MS [116]. *In vitro* recombinant TGF*β* decreased neuroantigen-specific T cell frequency and IFN*γ* and IL-4 producing T cells from MS patients [117]. 

More recently, others have demonstrated that murine Th17 cells can be generated by either IL-6 alone or IL-6 in combination with TGF*β*, but only those Th17 cells that had been generated with IL-6 alone are encephalitogenic [1]. IL-6 is increased in the CSF of other inflammatory neurological disorders including transverse myelitis (TM) and neuromyelitis optica (NMO) [118, 119], in the lesions of MS patients [120], and in some cases, correlates with relapse or general neurodegeneration in the progressive phase [121]. However, IL-6 is not increased in the CSF of MS patients and IL-6 levels do not correlate with other CSF parameters including oligoclonal bands, pleocytosis or IgG index [118, 122, 123]. Whether IL-6 is central to the generation of human Th17 cells remains under investigation [124, 125]. 

Even with confounding and often negative results of other studies in MS, it has been suggested to use the IL-6 receptor blocking antibody, Tocilizumab, as a therapeutic agent. Interestingly, IL-6 is increased after treatment of MS patients with IFN*β*, suggesting that this pleiotropic cytokine might not play an inflammatory pathogenic role in MS as IL-6 can have both systemic and local effects [126]. Because this cytokine differs in its necessity for EAE, it might represent an insurmountable task to go to trial due to the uncertain/unpredictable nature of the outcome without predictive animal models.

## 5. What Is the Effect of Immunotherapy on B Cell APC Function in MS?

As discussed earlier in this paper, T cells from RRMS patients treated with Rituximab had a poor response to antigen stimulation, suggesting that the lack of B cells in the periphery of these patients has a profound impact on T cell activation. Interestingly, circulating memory B cells are also reduced in RRMS patients during mitoxantrone therapy [96] and IFN*β* therapy [133]. IFN*β* treatment decreases MBP-elicited IFN*γ* and TNF*α*, but also decreases IL-10 and increases IL-6 [134]. Another study demonstrated that IFN*β* increases the expression of a phosphatase (SHP-1) that negatively regulates inflammatory T cell signaling from TNF*α*, IFN*γ*, IL-4, and IL-13 [135]. From these results, one may speculate that the effects of IFN*β* on T cells modulate their responsiveness to cytokine signals, some provided by B cells. On the other hand, *in vitro* studies show that Th17 cells and associated cytokines (IL-23, TGF*β* and IL-1*β*) were reduced, while anti-inflammatory cytokines produced by B cells and dendritic cells were increased. Reemerging B cells from RRMS patients treated with mitoxantrone therapy recovered the ability to produce IL-10 [107], but again, the impact of these IL-10-secreting B cells on neuroantigen-specific T cell responses was not tested.  Table 2 lists other current and emerging therapies and their possible impact on B cell function and emphasizes the urgent need to investigate how these drugs may affect B-T cell interactions in the patients receiving them.

In summary, the influence of B cells on T cell function is only beginning to be realized. Further investigations are required in order to fully comprehend the impact of current and emerging therapeutics on B cell responses, which in turn, may have profound impact on T cell function in autoimmune diseases such as MS.

## Figures and Tables

**Table 1:** Cytokines produced by B cells.

Cytokine	effects	Demonstration
IL-10	Inhibits T cell proliferation, IFN-*γ* and IL-2 production while enhancing Th2 responses [62–64]	Purified CD19+ peripheral human B cells produce IL-10 protein after dual stimulation with CpG and CD40L [65]. Maximal IL-10 production was noted with CD40L alone, compared to dual stimulation of BCR cross-link and CD40L [66]. IL-10 was secreted primarily by naïve CD19+CD27− B cells [39].
TGF-*β*	Induces tolerance and inflammation-dependent additional cytokine signals; IgA class switch and dampening NK activity [67–69]; development of Th17 and Treg cells [70]	Human total CD19+ B cells produce TGF-*β* mRNA in response to BCR cross-linking [71]. Anti-immunoglobulin treatment of murine B cell lymphomas induces active TGF-*β* [71].
LT-*α*	Required for the formation of germinal centers and follicles, upregulation of adhesion molecules [72, 73]	Purified human CD19+ B cells produce significant amounts of LT-*α* after dual stimulation with CpG and CD40L [65]. Maximal production was observed after dual stimulation of BCR cross-link and CD40L [66]. LT-*α* was secreted primarily by memory CD19+CD27+ B cells [39].
TNF-*α*	Increases IL-2 receptor and HLA-DR expression; induces T cell proliferation and IFN-*γ* production [74, 75]	Human CD19+ B cells produce TNF-*α* in response to CpG stimulation alone or in combination with CD40L [65, 76], or dual stimulation of BCR cross-link and CD40L [66]. TNF-*α* was secreted primarily by memory CD19+CD27+ B cells [39, 77].
IL-12	Critical for Th1 development, induces IFN*γ* production, enhances NK and CTL activity, inhibits Th2 development, and inhibits IgE class switching [78]	Human total CD19+ B cells produce IL-12p70 in response to dual stimulation with CpG and CD40L, but not in response to CpG (or CD40L) alone [65].
IL-6	Mediates early inflammation; activates endothelium and acts as growth and recruitment factor for lymphocytes [79–82]	Mediates early inflammation, activates endothelium, and acts as growth and recruitment factor for lymphocytes [79–81].
IFN-*γ*	Amplifies IFN-*γ* production; activates CTL and NK; critical for Th1 response [83, 84]	EBV-transformed B cell lines constitutively express IFN-*γ* as measured by qPCR [85]. PMA or IL-2 stimulation of EBV or B cell tumor lines induces IFN-*γ* [86].
IFN-*α*	Upregulates inflammatory cytokines [87]; implicated in the pathogenesis of several inflammatory autoimmune diseases including RA and IBD [88–90]	Purified human CD19+ B cells produce IFN-*α* transcripts in response to TLR8 agonist, but only in the first 24 hours post-stimulation [91]. Interestingly IFN-*β*, another type 1 interferon, is used as a treatment for RRMS.

**Table 2:** Possible impact of established and emerging therapies for MS on B cell function.

Therapeutic agent	Description	Impact on B-T interactions
Tysabri	Blocking antibody to *α*4*β*1 integrin (VLA4)	VLA4 is required for extravasation of lymphocytes across the blood brain barrier (BBB). A blockade of this adhesion molecule prevents autoreactive B and T cells from entering the CNS, thus limiting immune-mediated damage. CNS antigens are less available for antigen presentation without migration of antigen presenting cells (such as B cells) across the BBB.
MLN1202	CCR2 blocking antibody	Both B and T cells express CCR2, the receptor for MCP-1 [127]. Mouse studies show that MCP-1 is expressed in the microvessels [128] and that it is increased in MS [129]. This blocking antibody may abrogate leukocyte entrance into the CNS.
BAF312 and Fingolimod	blocking antibodies for the S1P receptor	These agents block egress of lymphocytes from secondary lymphoid organs. This therapy should decrease lymphocytes entry into the CNS. This mechanism is presumed to be similar to Tysabri.
Vitamin D3	Nutritional and environmental nutrient	The vitamin D3 receptor is expressed on GC and naïve and memory B cells [130]. Furthermore, Vitamin D3 reduces proliferative responses of B cells, reduces antibody secretion and class switching, inhibits maturation into memory and plasma phenotypes and induces apoptosis, but does not modulate the expression of HLA-DR or coreceptors [131]. Given these results, this therapy may have a profound effect on B cell activity in the context of MS.
CGP77116	Altered peptide ligand/mimetope for a dominant antigenic determinant of MBP (83–99)	CGP7716 will dampen the MBP-specific response by competing with native MBP. MBP-specific B cells could take up the APL and present it to T cells.
Glatiramer acetate (GA, Cop1, Copaxone)	Random polymer comprised of amino acids in a similar ratio as MBP	GA is known to skew the cytokine milieu toward a Th2 phenotype [132]. The effect of GA on B cell function is unknown.

## References

[1] Yang Y, Weiner J, Liu Y (2009). T-bet is essential for encephalitogenicity of both Th1 and Th17 cells. *Journal of Experimental Medicine*.

[2] Stromnes IM, Cerretti LM, Liggitt D (2008). Differential regulation of central nervous system autoimmunity by T(H)1 and T(H)17 cells. *Nature Medicine*.

[3] Sospedra M, Martin R (2005). Immunology of multiple sclerosis. *Annual Review of Immunology*.

[4] Naess A, Nyland H (1978). Multiple sclerosis. T lymphocytes in cerebrospinal fluid and blood. *European Neurology*.

[5] Hedegaard CJ, Krakauer M, Bendtzen K, Lund H, Sellebjerg F, Nielsen CH (2008). T helper cell type 1 (Th1), Th2 and Th17 responses to myelin basic protein and disease activity in multiple sclerosis. *Immunology*.

[6] El-behi M, Rostami A, Ciric B (2010). Current views on the roles of Th1 and Th17 cells in experimental autoimmune encephalomyelitis. *Journal of Neuroimmune Pharmacology*.

[7] Sandberg Wollheim M (1975). Immunoglobulin synthesis in vitro by cerebrospinal fluid cells in patients with meningoencephalitis of presumed viral origin. *Scandinavian Journal of Immunology*.

[8] Prineas JW, Wright RG (1978). Macrophages, lymphocytes, and plasma cells in the perivascular compartment in chronic multiple sclerosis. *Laboratory Investigation*.

[9] Lisak RP, Levinson AI, Zweiman B, Abdou NI (1975). T and B lymphocytes in multiple sclerosis. *Clinical and Experimental Immunology*.

[10] Holmøy T (2009). The discovery of oligoclonal bands: a 50-year anniversary. *European Neurology*.

[11] McFarland HF (2008). The B cell—old player, new position on the team. *The New England Journal of Medicine*.

[12] Hauser SL, Waubant E, Arnold DL (2008). B-cell depletion with rituximab in relapsing-remitting multiple sclerosis. *The New England Journal of Medicine*.

[13] Petereit HF, Moeller-Hartmann W, Reske D, Rubbert A (2008). Rituximab in a patient with multiple sclerosis—effect on B cells, plasma cells and intrathecal IgG synthesis. *Acta Neurologica Scandinavica*.

[14] Corinti S, Medaglini D, Prezzi C, Cavani A, Pozzi G, Girolomoni G (2000). Human dendritic cells are superior to B cells at presenting a major histocompatibility complex class II-restricted heterologous antigen expressed on recombinant Streptococcus gordonii. *Infection and Immunity*.

[15] Issekutz T, Chu E, Geha RS (1982). Antigen presentation by human B cells: T cell proliferation induced by Epstein Barr virus B lymphoblastoid cells. *The Journal of Immunology*.

[16] Good KL, Avery DT, Tangye SG (2009). Resting human memory B cells are intrinsically programmed for enhanced survival and responsiveness to diverse stimuli compared to naive B cells. *The Journal of Immunology*.

[17] Holmøy T, Vartdal F (2004). Cerebrospinal fluid T cells from multiple sclerosis patients recognize autologous Epstein-Barr virus-transformed B cells. *Journal of NeuroVirology*.

[18] Bar-Or A, Oliveira EML, Anderson DE (2001). Immunological memory: contribution of memory B cells expressing costimulatory molecules in the resting state. *Journal of Immunology*.

[19] Chan O, Madaio MP, Shlomchik MJ (1997). The roles of B cells in MRL/lpr murine lupus. *Annals of the New York Academy of Sciences*.

[20] Chan O, Madaio MP, Shlomchik MJ (1997). The roles of B cells in MRL/lpr murine lupus. *Annals of the New York Academy of Sciences*.

[21] Crawford A, MacLeod M, Schumacher T, Corlett L, Gray D (2006). Primary T cell expansion and differentiation in vivo requires antigen presentation by B cells. *Journal of Immunology*.

[22] O’Neill SK, Shlomchik MJ, Glant TT, Cao Y, Doodes PD, Finnegan A (2005). Antigen-specific B cells are required as APCs and autoantibody-producing cells for induction of severe autoimmune arthritis. *Journal of Immunology*.

[23] Wong FS, Wen LI, Tang M (2004). Investigation of the role of B-cells in type 1 diabetes in the NOD mouse. *Diabetes*.

[24] Evans DE, Munks MW, Purkerson JM, Parker DC (2000). Resting B lymphocytes as APC for naive T lymphocytes: dependence on CD40 ligand/CD40. *Journal of Immunology*.

[25] Krieger JI, Grammer SF, Grey HM, Chesnut RW (1985). Antigen presentation by splenic B cells: resting B cells are ineffective, whereas activated B cells are effective accessory cells for T cell responses. *Journal of Immunology*.

[26] Rock KL, Benacerraf B, Abbas AK (1984). Antigen presentation by hapten-specific B lymphocytes. I. Role of surface immunoglobulin receptors. *Journal of Experimental Medicine*.

[27] Jiang W, Lederman MM, Harding CV, Rodriguez B, Mohner RJ, Sieg SF (2007). TLR9 stimulation drives naïve B cells to proliferate and to attain enhanced antigen presenting function. *European Journal of Immunology*.

[28] Harp CT, Lovett-Racke AE, Racke MK, Frohman EM, Monson NL (2008). Impact of myelin-specific antigen presenting B cells on T cell activation in multiple sclerosis. *Clinical Immunology*.

[29] von Bergwelt-Baildon M, Schultze JL, Maecker B (2004). Correspondence re R. Lapointe et al., CD40-stimulated B lymphocytes pulsed with tumor antigens are effective antigen-presenting cells that can generate specific T cells. *Cancer Research*.

[30] Lapointe R, Bellemare-Pelletier A, Housseau F, Thibodeau J, Hwu P (2003). CD40-stimulated B lymphocytes pulsed with tumor antigens are effective antigen-presenting cells that can generate specific T cells. *Cancer Research*.

[31] Von Bergwelt-Baildon M, Shimabukuro-Vornhagen A, Popov A (2006). CD40-activated B cells express full lymph node homing triad and induce T-cell chemotaxis: potential as cellular adjuvants. *Blood*.

[32] Fuchs EJ, Matzinger P (1992). B cells turn off virgin but not memory T cells. *Science*.

[33] Chen LC, Delgado JC, Jensen PE, Chen X (2009). Direct expansion of human allospecific FoxP3+CD4+ regulatory T cells with allogeneic B cells for therapeutic application. *Journal of Immunology*.

[34] Eynon EE, Parker DC (1992). Small B cells as antigen-presenting cells in the induction of tolerance to soluble protein antigens. *Journal of Experimental Medicine*.

[35] Reichardt P, Dornbach B, Rong S (2007). Naive B cells generate regulatory T cells in the presence of a mature immunologic synapse. *Blood*.

[36] Attanavanich K, Kearney JF (2004). Marginal zone, but not follicular B cells, are potent activators of naive CD4 T cells. *Journal of Immunology*.

[37] Kakiuchi T, Chesnut RW, Grey HM (1983). B cells as antigen-presenting cells: the requirement for B cell activation. *Journal of Immunology*.

[38] Pasare C, Morafo V, Entringer M (1998). Presence of activated antigen-binding B cells during immunization enhances relative levels of IFN-*γ* in T cell responses. *Journal of Immunology*.

[39] Harp CT, Ireland S, Davis LS (2010). Memory B cells from a subset of treatment-naive relapsing-remitting multiple sclerosis patients elicit CD4(+) T-cell proliferation and IFN-*γ*
production in response to myelin basic protein and myelin oligodendrocyte glycoprotein. *European Journal of Immunology*.

[40] Honjo T, Alt FW, Neuberger MS (2004). *Molecular Biology of B cells*.

[41] Yanaba K, Bouaziz JD, Matsushita T, Magro CM, Clair ST, Tedder TF (2008). B-lymphocyte contributions to human autoimmune disease. *Immunological Reviews*.

[42] Dyment DA, Ebers GC, Sadovnick AD (2004). Genetics of multiple sclerosis. *Lancet Neurology*.

[43] Perrin PJ, June CH, Maldonado JH, Ratts RB, Racke MK (1999). Blockade of CD28 during in vitro activation of encephalitogenic T cells or after disease onset ameliorates experimental autoimmune encephalomyelitis. *Journal of Immunology*.

[44] Chen L (2004). Co-inhibitory molecules of the B7-CD28 family in the control of T-cell immunity. *Nature Reviews Immunology*.

[45] Stuart RW, Racke MK (2002). Targeting T cell costimulation in autoimmune disease. *Expert Opinion on Therapeutic Targets*.

[46] Salomon B, Bluestone JA (2001). Complexities of CD28/B7: CTLA-4 costimulatory pathways in autoimmunity and transplantation. *Annual Review of Immunology*.

[47] Genç K, Dona DL, Reder AT (1997). Increased CD80(+) B cells in active multiple sclerosis and reversal by interferon *β*-1b therapy. *Journal of Clinical Investigation*.

[48] Amu S, Tarkowski A, Dörner T, Bokarewa M, Brisslert M (2007). The human immunomodulatory CD25+ B cell population belongs to the memory B cell pool. *Scandinavian Journal of Immunology*.

[49] Lovett-Racke AE, Trotter JL, Lauber J, Perrin PJ, June CH, Racke MK (1998). Decreased dependence of myelin basic protein-reactive T cells on CD28- mediated costimulation in multiple sclerosis patients. *Journal of Clinical Investigation*.

[50] Comabella M, Craig DW, Carmiña-Tato M (2008). Identification of a novel risk locus for multiple sclerosis at 13q31.3 by a pooled genome-wide scan of 500,000 single nucleotide polymorphisms. *PLoS ONE*.

[51] Dyment DA, Herrera BM, Cader MZ (2005). Complex interactions among MHC haplotypes in multiple sclerosis: susceptibility and resistance. *Human Molecular Genetics*.

[52] Hafler DA, Compston A, Sawcer S (2007). Risk alleles for multiple sclerosis identified by a genomewide study. *The New England Journal of Medicine*.

[53] Bishop GA, Hostager BS (2003). The CD40-CD154 interaction in B cell-T cell liaisons. *Cytokine and Growth Factor Reviews*.

[54] Boumpas DT, Furie R, Manzi S (2003). A short course of BG9588 (anti-CD40 ligand antibody) improves serologic activity and decreases hematuria in patients with proliferative lupus glomerulonephritis. *Arthritis and Rheumatism*.

[55] Mirabet M, Barrabés JA, Quiroga A, Garcia-Dorado D (2008). Platelet pro-aggregatory effects of CD40L monoclonal antibody. *Molecular Immunology*.

[56] Levesque MC (2009). Translational mini-review series on B cell-directed therapies: recent advances in B cell-directed biological therapies for autoimmune disorders. *Clinical and Experimental Immunology*.

[57] Carpenter EL, Mick R, Rüter J, Vonderheide RH (2009). Activation of human B cells by the agonist CD40 antibody CP-870,893 and augmentation with simultaneous toll-like receptor 9 stimulation. *Journal of Translational Medicine*.

[58] Gantner F, Hermann P, Nakashima K, Matsukawa S, Sakai K, Bacon KB (2003). CD40-dependent and -independent activation of human tonsil B cells by CpG oligodeoxynucleotides. *European Journal of Immunology*.

[59] Wang Y, Li M, Song M (2008). Expression of OX40 ligand in microglia activated by IFN-*γ* sustains a protective CD4+ T-cell response in vitro. *Cellular Immunology*.

[60] Ito T, Wang YH, Duramad O (2006). 0X40 ligand shuts down IL-10-producing regulatory T cells. *Proceedings of the National Academy of Sciences of the United States of America*.

[61] Li J, Li LI, Shang X (2008). Negative regulation of IL-17 production by OX40/OX40L interaction. *Cellular Immunology*.

[62] Taga K, Tosato G (1992). IL-10 inhibits human T cell proliferation and IL-2 production. *Journal of Immunology*.

[63] Moore KW, de Waal Malefyt R, Coffman RL (1993). Interleukin-10. *Annual Review of Immunology*.

[64] Moore KW, de Waal Malefyt R, Coffman RL (2001). Interleukin-10 and the interleukin-10 receptor. *Annual Review of Immunology*.

[65] Wagner M, Poeck H, Jahrsdoerfer B (2004). IL-12p70-dependent Th1 induction by human B cells requires combined activation with CD40 ligand and CpG DNA. *Journal of Immunology*.

[66] Duddy ME, Alter A, Bar-Or A (2004). Distinct profiles of human B cell effector cytokines: a role in immune regulation?. *Journal of Immunology*.

[67] Rubtsov YP, Rudensky AY (2007). TGF*β* signalling in control of T-cell-mediated self-reactivity. *Nature Reviews Immunology*.

[68] Li MO, Wan YY, Sanjabi S, Robertson AKL, Flavell RA (2006). Transforming growth factor-*β* regulation of immune responses. *Annual Review of Immunology*.

[69] Veldhoen M, Stockinger B (2006). TGF*β*1, a “Jack of all trades”: the link with pro-inflammatory IL-17-producing T cells. *Trends in Immunology*.

[70] Bettelli E, Oukka M, Kuchroo VK (2007). T(H)-17 cells in the circle of immunity and autoimmunity. *Nature Immunology*.

[71] Warner GL, Ludlow JW, Nelson DA, Gaur A, Scott DW (1992). Anti-immunoglobulin treatment of murine B-cell lymphomas induces active transforming growth factor beta but pRB hypophosphorylation is transforming growth factor beta independent. *Cell Growth &amp; Differentiation*.

[72] Ruddle NH (1999). Lymphoid neo-organogenesis: lymphotoxin’s role in inflammation and development. *Immunologic Research*.

[73] Pfeffer K (2003). Biological functions of tumor necrosis factor cytokines and their receptors. *Cytokine & Growth Factor Reviews*.

[74] Apostolaki M, Armaka M, Victoratos P, Kollias G (2010). Cellular mechanisms of TNF function in models of inflammation and autoimmunity. *Current Directions in Autoimmunity*.

[75] Kollias G, Douni E, Kassiotis G, Kontoyiannis D (1999). On the role of tumor necrosis factor and receptors in models of multiorgan failure, rheumatoid arthritis, multiple sclerosis and inflammatory bowel disease. *Immunological Reviews*.

[76] Scheurich P, Thoma B, Ucer U, Pfizenmaier K (1987). Immunoregulatory activity of recombinant human tumor necrosis factor (TNF)-*α*: induction of TNF receptors on human T cells and TNF-*α*-mediated enhancement of T cell responses. *Journal of Immunology*.

[77] Harp CT, Ireland S, Davis LS (2010). Memory B cells from a subset of treatment-naive relapsing-remitting multiple sclerosis patients elicit CD4(+) T-cell proliferation and IFN-gamma production in response to myelin basic protein and myelin oligodendrocyte glycoprotein. *European Journal of Immunology*.

[78] Trinchieri G (1994). Interleukin-12: a cytokine produced by antigen-presenting cells with immunoregulatory functions in the generation of T-helper cells type 1 and cytotoxic lymphocytes. *Blood*.

[79] Romano M, Sironi M, Toniatti C (1997). Role of IL-6 and its soluble receptor in induction of chemokines and leukocyte recruitment. *Immunity*.

[80] Van Snick J (1990). Interleukin-6: an overview. *Annual Review of Immunology*.

[81] Kimura A, Kishimoto T (2010). IL-6: regulator of Treg/Th17 balance. *European Journal of Immunology*.

[82] Hirano T, Akira S, Taga T, Kishimoto T (1990). Biological and clinical aspects of interleukin 6. *Immunology Today*.

[83] Billiau A, Matthys P (2009). Interferon-*γ*: a historical perspective. *Cytokine and Growth Factor Reviews*.

[84] O’Garra A, Steinman L, Gijbels K (1997). CD4+ T-cell subsets in autoimmunity. *Current Opinion in Immunology*.

[85] Dayton MA, Knobloch TJ, Benjamin D (1992). Human B cell lines express the interferon gamma gene. *Cytokine*.

[86] Pang Y, Norihisa Y, Benjamin D, Kantor RRS, Young HA (1992). Interferon-*γ* gene expression in human B-cell lines: induction by interleukin-2, protein kinase C activators, and possible effect of hypomethylation on gene regulation. *Blood*.

[87] Theofilopoulos AN, Baccala R, Beutler B, Kono DH (2005). Type I interferons (*α*/*β*) in immunity and autoimmunity. *Annual Review of Immunology*.

[88] Arend WP (2002). The balance between IL-1 and IL-1Ra in disease. *Cytokine and Growth Factor Reviews*.

[89] Fontana A, Hengartner H, Weber E (1982). Interleukin 1 activity in the synovial fluid of patients with rheumatoid arthritis. *Rheumatology International*.

[90] Isaacs KL, Sartor RB, Haskill S (1992). Cytokine messenger RNA profiles in inflammatory bowel disease mucosa detected by polymerase chain reaction amplification. *Gastroenterology*.

[91] Hanten JA, Vasilakos JP, Riter CL (2008). Comparison of human B cell activation by TLR7 and TLR9 agonists. *BMC Immunology*.

[92] Anderton SM, Fillatreau S (2008). Activated B cells in autoimmune diseases: the case for a regulatory role. *Nature Clinical Practice Rheumatology*.

[93] Fillatreau S, Sweenie CH, McGeachy MJ, Gray D, Anderton SM (2002). B cells regulate autoimmunity by provision of IL-10. *Nature Immunology*.

[94] Mann MK, Maresz K, Shriver LP, Tan Y, Dittel BN (2007). B cell regulation of CD4+CD25+ T regulatory cells and IL-10 via B7 is essential for recovery from experimental autoimmune encephalomyelitis. *Journal of Immunology*.

[95] Correale J, Farez M, Razzitte G (2008). Helminth infections associated with multiple sclerosis induce regulatory B cells. *Annals of Neurology*.

[96] Duddy M, Niino M, Adatia F (2007). Distinct effector cytokine profiles of memory and naive human B cell subsets and implication in multiple sclerosis. *Journal of Immunology*.

[97] D’Cruz DP, Mellor-Pita S, Joven B (2004). Transverse myelitis as the first manifestation of systemic lupus erythematosus or lupus-like disease: good functional outcome and relevance of antiphospholipid antibodies. *Journal of Rheumatology*.

[98] Heo YUJ, Joo YB, Oh HJ (2010). IL-10 suppresses Th17 cells and promotes regulatory T cells in the CD4+ T cell population of rheumatoid arthritis patients. *Immunology Letters*.

[99] Ware CF, VanArsdale TL, Crowe PD, Browning JL (1995). The ligands and receptors of the lymphotoxin system. *Current Topics in Microbiology and Immunology*.

[100] Locksley RM, Killeen N, Lenardo MJ (2001). The TNF and TNF receptor superfamilies: integrating mammalian biology. *Cell*.

[101] Fu YX, Molina H, Matsumoto M, Huang G, Min J, Chaplin DD (1997). Lymphotoxin-*α* (LT*α*) supports development of splenic follicular structure that is required for IgG responses. *Journal of Experimental Medicine*.

[102] Yu P, Wang Y, Chin RK (2002). B cells control the migration of a subset of dendritic cells into B cell follicles via CXC chemokine ligand 13 in a lymphotoxin-dependent fashion. *Journal of Immunology*.

[103] Fu YX, Huang G, Wang Y, Chaplin DD (2000). Lymphotoxin-*α*-dependent spleen microenvironment supports the generation of memory B cells and is required for their subsequent antigen-induced activation. *Journal of Immunology*.

[104] Fu YX, Huang G, Wang Y, Chaplin DD (1998). B lymphocytes induce the formation of follicular dendritic cell clusters in a lymphotoxin *α*-dependent fashion. *Journal of Experimental Medicine*.

[105] Ware CF, Crowe PD, Vanarsdale TL (1991). Tumor necrosis factor (TNF) receptor expression in T lymphocytes: differential regulation of the type I TNF receptor during activation of resting and effector T cells. *Journal of Immunology*.

[106] Plant SR, Aknett HA, Ting JPY (2005). Astroglial-derived lymphotoxin-*α* exacerbates inflammation and demyelination, but not remyelination. *Glia*.

[107] Bar-Or A, Fawaz L, Fan B (2010). Abnormal B-cell cytokine responses a trigger of T-cell-mediated disease in MS?. *Annals of Neurology*.

[108] Pistoia V (1997). Production of cytokines by human B cells in health and disease. *Immunology Today*.

[109] Weaver CT, Harrington LE, Mangan PR, Gavrieli M, Murphy KM (2006). Th17: an effector CD4 T cell lineage with regulatory T cell ties. *Immunity*.

[110] Kikly K, Liu L, Na S, Sedgwick JD (2006). The IL-23/Th axis: therapeutic targets for autoimmune inflammation. *Current Opinion in Immunology*.

[111] McGeachy MJ, Bak-Jensen KS, Chen YI (2007). TGF-*β* and IL-6 drive the production of IL-17 and IL-10 by T cells and restrain T-17 cell-mediated pathology. *Nature Immunology*.

[112] Bettelli E, Carrier Y, Gao W (2006). Reciprocal developmental pathways for the generation of pathogenic effector T17 and regulatory T cells. *Nature*.

[113] Veldhoen M, Hocking RJ, Atkins CJ, Locksley RM, Stockinger B (2006). TGF*β* in the context of an inflammatory cytokine milieu supports de novo differentiation of IL-17-producing T cells. *Immunity*.

[114] Jäger A, Dardalhon V, Sobel RA, Bettelli E, Kuchroo VK (2009). Th1, Th17, and Th9 effector cells induce experimental autoimmune encephalomyelitis with different pathological phenotypes. *Journal of Immunology*.

[115] Racke MK, Cannella B, Albert P, Sporn M, Raine CS, McFarlin DE (1992). Evidence of endogenous regulatory function of transforming growth factor-*β*1 in experimental allergic encephalomyelitis. *International Immunology*.

[116] Calabresi PA, Fields NS, Maloni HW (1998). Phase 1 trial of transforming growth factor beta 2 in chronic progressive MS. *Neurology*.

[117] Link J, He B, Navikas V (1995). Transforming growth factor-*β*1 suppresses autoantigen-induced expression of pro-inflammatory cytokines but not of interleukin-10 in multiple sclerosis and myasthenia gravis. *Journal of Neuroimmunology*.

[118] Uzawa A, Mori M, Ito M (2009). Markedly increased CSF interleukin-6 levels in neuromyelitis optica, but not in multiple sclerosis. *Journal of Neurology*.

[119] Kaplin AI, Deshpande DM, Scott E (2005). IL-6 induces regionally selective spinal cord injury in patients with the neuroinflammatory disorder transverse myelitis. *Journal of Clinical Investigation*.

[120] Lock C, Hermans G, Pedotti R (2002). Gene-microarray analysis of multiple sclerosis lesions yields new targets validated in autoimmune encephalomyelitis. *Nature Medicine*.

[121] Maimone D, Gregory S, Arnason BGW, Reder AT (1991). Cytokine levels in the cerebrospinal fluid and serum of patients with multiple sclerosis. *Journal of Neuroimmunology*.

[122] Hauser SL, Doolittle TH, Lincoln R, Brown RH, Dinarello CA (1990). Cytokine accumulations in CSF of multiple sclerosis patients: frequent detection of interleukin-1 and tumor necrosis factor but not interleukin-6. *Neurology*.

[123] Laurenzi MA, Siden A, Persson MAA, Norkrans G, Hagberg L, Chiodi F (1990). Cerebrospinal fluid interleukin-6 activity in HIV infection and inflammatory and noninflammatory diseases of the nervous system. *Clinical Immunology and Immunopathology*.

[124] Benwell RK, Lee DR (2010). Essential and synergistic roles of IL1 and IL6 in human Th17 differentiation directed by TLR ligand-activated dendritic cells. *Clinical Immunology*.

[125] Lim HW, Lee J, Hillsamer P, Kim CH (2008). Human Th17 cells share major trafficking receptors with both polarized effector T cells and FOXP3+ regulatory T cells. *Journal of Immunology*.

[126] Nakatsuji Y, Nakano M, Moriya M (2006). Beneficial effect of interferon-*β* treatment in patients with multiple sclerosis is associated with transient increase in serum IL-6 level in response to interferon-*β* injection. *Cytokine*.

[127] Frade JMR, Mellado M, Del Real G, Gutierrez-Ramos JC, Lind P, Martinez-A. C. C (1997). Characterization of the CCR2 chemokine receptor: functional CCR2 receptor expression in B cells. *Journal of Immunology*.

[128] Dzenko KA, Andjelkovic AV, Kuziel WA, Pachter JS (2001). The chemokine receptor CCR2 mediates the binding and internalization of monocyte chemoattractant protein-1 along brain microvessels. *Journal of Neuroscience*.

[129] Nakajima H, Sugino M, Kimura F (2007). Increased intrathecal chemokine receptor CCR2 expression in multiple sclerosis. *Biomark Insights*.

[130] Morgan JW, Kouttab N, Ford D, Maizel AL (2000). Vitamin D-mediated gene regulation in phenotypically defined human B cell subpopulations. *Endocrinology*.

[131] Chen S, Sims GP, Xiao XC, Yue YG, Chen S, Lipsky PE (2007). Modulatory effects of 1,25-dihydroxyvitamin D on human B cell differentiation. *Journal of Immunology*.

[132] Neuhaus O, Farina C, Yassouridis A (2000). Multiple sclerosis: comparison of copolymer-1-reactive T cell lines from treated and untreated subjects reveals cytokine shift from T helper 1 to T helper 2 cells. *Proceedings of the National Academy of Sciences of the United States of America*.

[133] Niino M, Hirotani M, Miyazaki Y, Sasaki H (2009). Memory and naïve B-cell subsets in patients with multiple sclerosis. *Neuroscience Letters*.

[134] Hedegaard CJ, Krakauer M, Bendtzen K, Sørensen PS, Sellebjerg F, Nielsen CH (2008). The effect of *β*-interferon therapy on myelin basic protein-elicited CD4+ T cell proliferation and cytokine production in multiple sclerosis. *Clinical Immunology*.

[135] Christophi GP, Panos M, Hudson CA (2009). Interferon-*β* treatment in multiple sclerosis attenuates inflammatory gene expression through inducible activity of the phosphatase SHP-1. *Clinical Immunology*.

